# Supplementary material for: Organoids transplantation attenuates intestinal ischemia/reperfusion injury in mice through L-Malic acid-mediated M2 macrophage polarization
Source: Nat Commun. 2023 Oct 25;14:6779. doi: 10.1038/s41467-023-42502-0 (PMC10600233; doi:10.1038/s41467-023-42502-0)
Supplement: Supplementary file 1 — Supplementary Information File [file 41467_2023_42502_MOESM1_ESM.pdf]

Supplementary Information for

Organoids transplantation attenuates intestinal ischemia/reperfusion injury in mice through L-malic acid-mediated M2 macrophage polarization

List of all Authors: Fang-Ling Zhang *et al*.

*Corresponding author: Ke-Xuan Liu. Email: liukexuan705@163.com

Supplementary Figures and figure legends

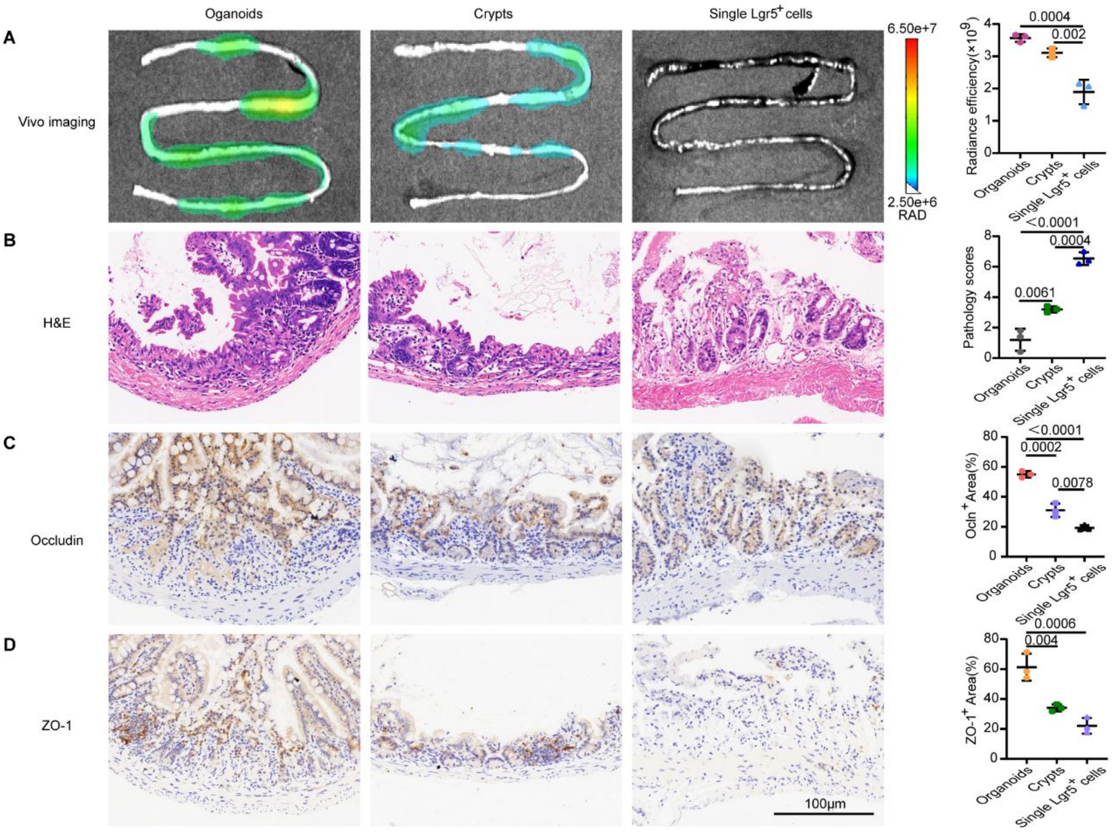

Supplementary Figure 1. Analysis the treatment efficacy of three alternative grafting materials. Related to Figure 1.

(A) Ex vivo imaging of transplanted organoids, fresh isolated crypts, and sorted LGR5$^+$ stem cells excised from transplanted mice at 36 h and the quantification of the fluorescence signal (n=3 mice/group). (B) Representative H & E images and the analysis of pathology scores in transplanted organoids, fresh isolated crypts, and sorted LGR5$^+$ stem cells excised from transplanted mice at 36 h after intestinal I/R (n=3 mice/group). (C) Representative immunohistochemistry images and quantification of the area of Occludin staining in transplanted organoids, fresh isolated crypts, and sorted LGR5$^+$ stem cells excised from transplanted mice at 36 h after intestinal I/R (n=3 mice/group). (D) Representative immunohistochemistry

images and quantification of the area of ZO-1 staining in transplanted organoids, fresh isolated crypts, and sorted $LGR5^+$ stem cells excised from transplanted mice at 36 h after intestinal I/R (n=3 mice/group). Scale bar, 100 μm. The statistical tests employed included: one-way ANOVA followed by the Tukey test for multiple comparisons. *P < 0.05, ** P < 0.01, *** P < 0.001, **** P < 0.0001. Each dot represents data from a single mouse (A-D). Bar graphs represent mean ± standard deviation (SD). Source data are provided as a Source Data file.

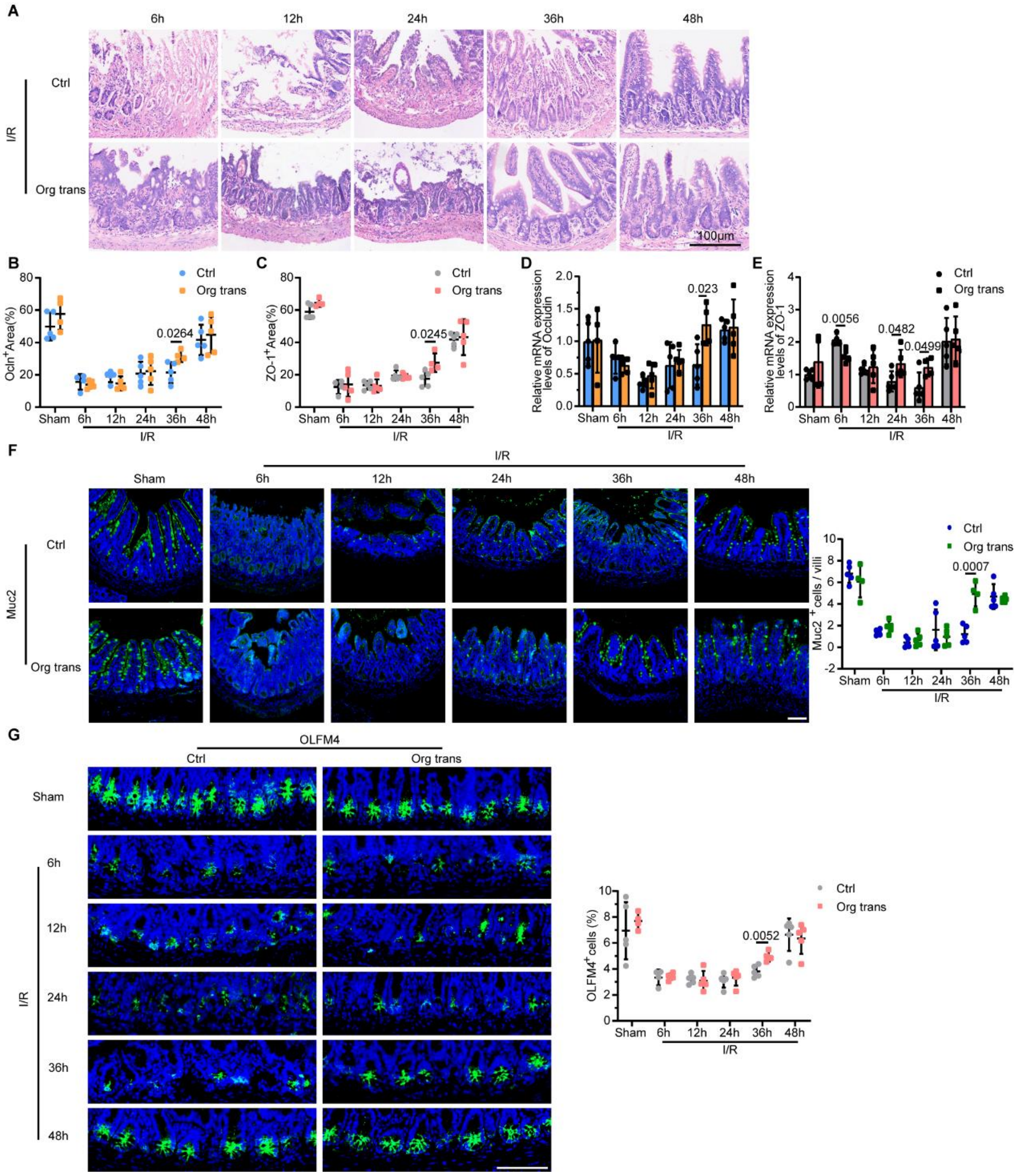

Supplementary Figure 2. Organoids transplantation contribute to promote mucosal recovery and self-renewal. Related to Figure 1.

(A) Representative images of Hematoxylin and eosin (H & E) staining of small intestinal tissue. (B) Quantification of the area of Occludin immunohistochemistry staining. (C) Quantification of the area of ZO-1 immunohistochemistry staining (n = 4 mice for sham group with organoids transplanted, control group at 6 h and transplanted group at 36 h, n = 5 mice for the rest groups). (D and E) qRT-PCR

analysis of Occludin (C) and ZO-1 (D) mRNA in small intestinal tissues from mice at indicated time points after intestinal I/R (n = 4 mice for sham group with organoids transplanted, control group at 6 h and transplanted group at 36 h, n = 5 mice for the rest groups). (F) Immunofluorescence image of the intestinal villus following Muc2 staining and quantification of $Muc2^+$ goblet cells (n = 4 mice for sham group with organoids transplanted, control group at 6 h and transplanted group at 36 h, n = 5 mice for the rest groups). (G) Immunofluorescence image of intestinal crypts following $OLFM4^+$ staining and quantification of the mean immunofluorescence area of $OLFM4^+$ cells (n = 4 mice for sham group with organoids transplanted, control group at 6 h and transplanted group at 36 h, n = 5 mice for the rest groups). Twenty-five villus or crypts from each mouse were obtained for each group. Scale bar, 100 μm. The statistical tests employed included: two-tailed student's t-test and Mann-Whitney test. *P < 0.05, ** P < 0.01, *** P < 0.001. Each dot represents data from a single mouse (B-G). Bar graphs represent mean ± SD. Source data are provided as a Source Data file.

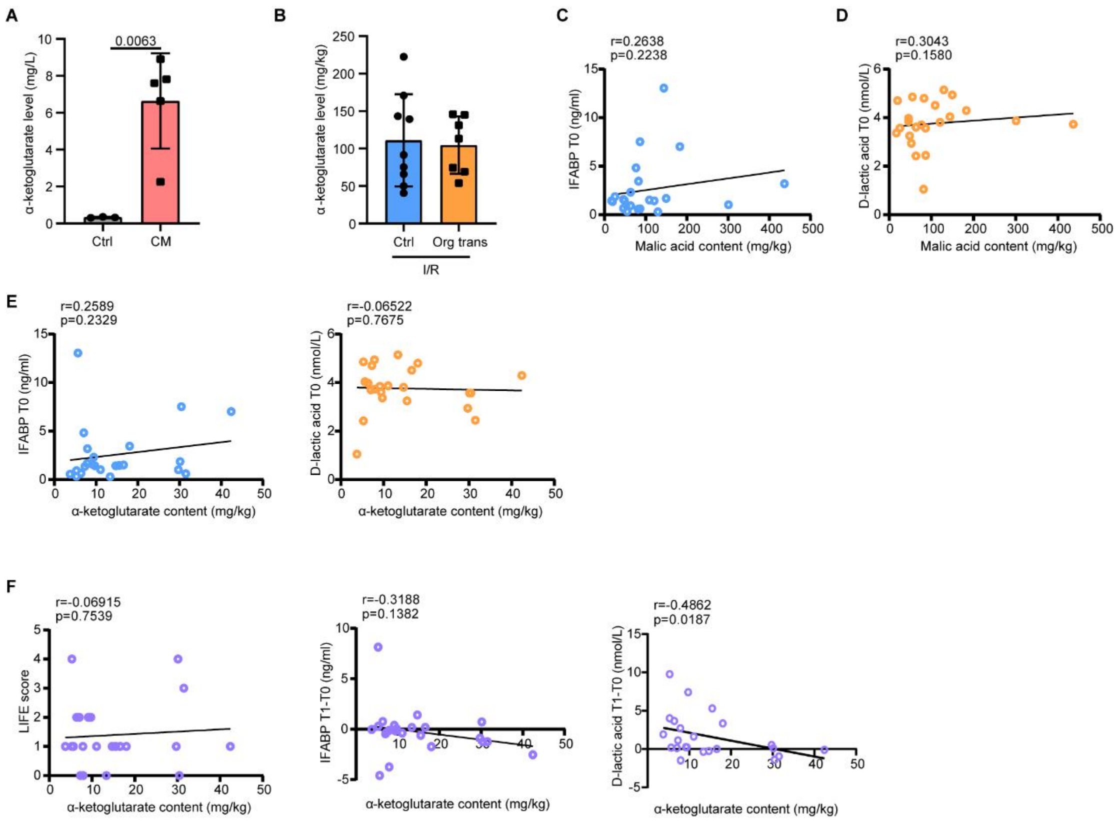

Supplementary Figure 3. Relationship between TCA metabolites and postoperative intestinal injury in patients undergoing cardiopulmonary bypass surgery. Related to Figure 4.

(A) α-ketoglutarate levels in organoid-derived conditioned and control medium groups using targeted LC-MS metabolomic analysis (n = 3 biological replicates for control group, n = 5 biological replicates for CM group). (B) α-ketoglutarate levels in the cecal contents of the organoid-transplanted and control group 36 h after I/R using targeted LC-MS metabolomic analysis (n = 7 mice for control group, n = 9 mice for transplanted group). (C) Correlation analysis between preoperative fecal MA levels and serum I-FABP levels in patients at T0 (n = 23 samples/group). (D) Correlation analysis between preoperative fecal MA levels and serum D-lactate levels in patients at T0 (n = 23 samples/group). (E) Correlation analysis between preoperative fecal

α-ketoglutarate levels and serum I-FABP and D-lactate levels in patients at T0 (n = 23 samples/group). (F) Correlation analysis between preoperative patient fecal α-ketoglutarate content and LIFE score, serum I-FABP, and D-lactate levels in patients at T1 compared to T0 (n = 23 samples/group). The statistical employed included: two-tailed student's t-test and Spearman's correlation coefficients. *P < 0.05, ** P < 0.01. Each dot represents data from a single sample (A-F). Bar graphs represent mean ± SD. Source data are provided as a Source Data file.

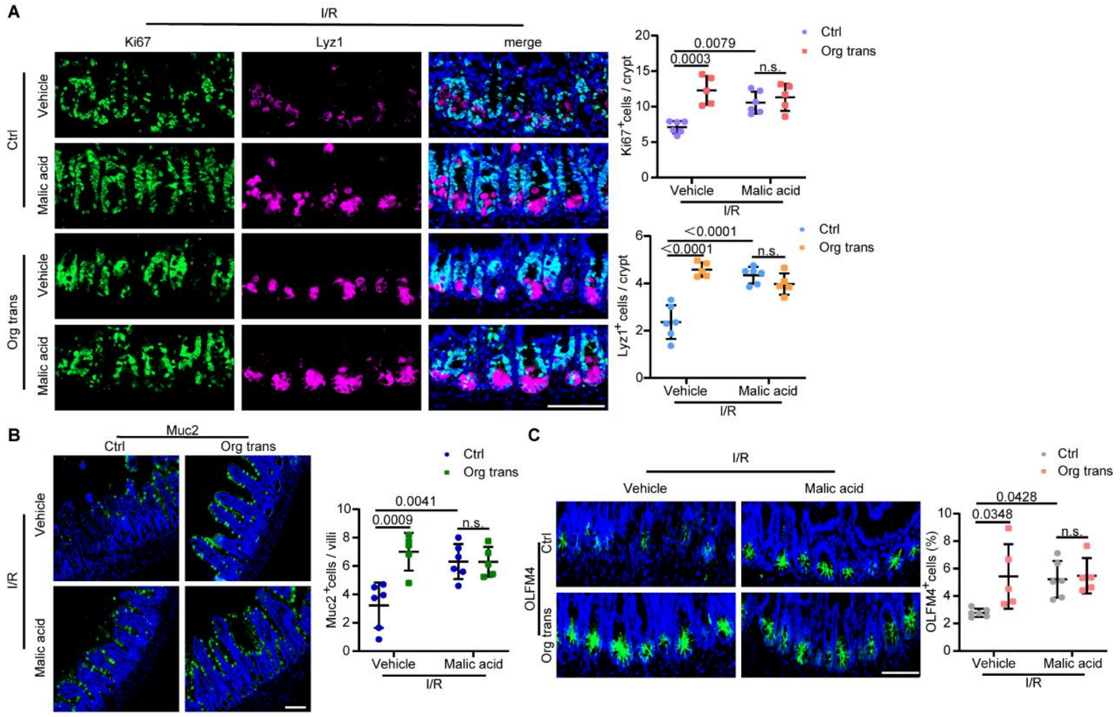

Supplementary Figure 4. Administration of MA contribute to promote mucosal recovery and self-renewal. Related to Figure 5.

(A) Immunofluorescence image of intestinal crypts following Ki67^{+}, Lyz1^{+} staining and quantification of Ki67^{+}, Lyz1^{+} Paneth cells (n = 5 mice for transplanted groups, n = 6 mice for the rest groups). (B) Immunofluorescence image of the intestinal villus following Muc2 staining and quantification of Muc2^{+} goblet cells (n = 5 mice for transplanted groups, n = 6 mice for the rest groups). (C) Immunofluorescence image of intestinal crypts following OLFM4^{+} staining and quantification of the mean immunofluorescence area of OLFM4^{+} cells (n = 5 mice for transplanted groups, n = 6 mice for the rest groups). Twenty-five crypts or villus from each mouse were obtained for each group. Scale bar, 100 μm. The statistical tests employed included: two-way ANOVA followed by the Tukey test for multiple comparisons. *$P < 0.05$, ** $P < 0.01$,

*** P < 0.001, **** P < 0.0001. Each dot represents data from a single mouse (A-C).

Bar graphs represent mean ± SD. Source data are provided as a Source Data file.

Supplementary Table 1. Mouse primers used for qPCR analysis (5’ to 3').

Related to Figure 1 to Figure 7 and supplemental Figure 2.

Gene	forward	reverse
18s	AGTCCCTGCCCTTTGTACACA	CGATCCGAGGGCCTCACTA
Il-10	GCCACATGCTCCTAGAGCTG	CAGCTGGTCCTTTGTTTGAAA
Ki67	ACCGTGGAGTAGTTTATCTGGG	TGTTTCCAGTCCGCTTACTTCT
Lysozyme	GAGACCGAAGCACCGACTATG	CGGTTTTGACATTGTGTTCGC
CD206	GCGCTGCGTGGACGCTCTAA	ACAGGGTGACGGAAGCCCAGT
ZO-1	TGCAATTCCAAATCCAAACC	AGAGACAAGATGTCCGCCAG
Occludin	GGACTGTCAACTCTTTCCGC	CATTTATGATGAACAGCCCC
Il-1β	GGTCAAAGGTTTGGAAGCAG	TGTGAAATGCCACCTTTTGA
Il-6	ACCAGAGGAAATTTTCAATAGGC	TGATGCACTTGCAGAAAACA
TNF-α	AGGGTCTGGGCCATAGAACT	CCACCACGCTCTTCTGTCTAC
CD86	TGCTCATCATTGTATGTCAC	GTCTCTCTGTCAGCGTTACT
Ym1/2	TCACAGGTCTGGCAATTCTTCTG	TTTGTCCTTAGGAGGGCTTCCTC
Arg1	CAGTTGGAAGCATCTCTGGC	GTGAGCATCCACCCAAATGAC
SOCS1	ATGGTAGCACGCAACCAGGTG	CTCCAGCAGCTCGAAAAGGCA
SOCS2	GGAAGTATGACTGTTAATGAAGCC	CCCAGATCGTACCGGTACATT
SOCS3	GCTCCAAAAGCGAGTACCAG	GGATGCGTAGGTTCTTGGTC
SOCS4	CCTCGCTCAGATTTAGCCTTTAG	TGGAACAAGGCAGTGGACGTA
SOCS5	GCCTTACAGCTGGGACTGAG	AGTGGCTTTGACTGCTTGCT
SOCS6	CTCTCACCATTGCTACCTCCAA	AGAGTCCCTGATTGAATGCTCGAT

SOCS7	GAATTGCTCCCATCAGAGCGTCA	GTTCTTGGAGTGCATGATGGCTCT

Supplementary Table 2. Flow cytometry antibodies.

ANTIBODIES	SOURCE	IDENTIFIER	DILUTION
Anti-mouse CD16/32, clone 2.4G2	BD Biosciences	Cat# 553141	1 µg per 10^6 cells in 100 µl volume
Anti-mouse CD11b, BV510, clone M1/70	Biolegend	Cat# 101263	0.4 µg per 10^6 cells in 100 µl volume
Anti-mouse CD11b, PE, clone M1/70	Biolegend	Cat# 101208	0.25 µg per 10^6 cells in 100 µl volume
Anti-mouse Ly-6G, PerCP/Cy5.5, clone 1A8	Biolegend	Cat# 127615	0.25 µg per 10^6 cells in 100 µl volume
Anti-mouse CD45, APC-Cy7, clone I3/2.3	Thermo Fisher Scientifific	Cat# A15395	0.2 µg per 10^6 cells in 100 µl volume
Anti-mouse Ly-6C, PE-Cy7, clone HK1.4	Biolegend	Cat# 128017	0.06 µg per 10^6 cells in 100 µl volume
Anti-mouse CD206, APC, clone C068C2	Biolegend	Cat# 141707	0.5 µg per 10^6 cells in 100 µl volume
Anti-mouse IL10, PE, clone JES5-16E3	Biolegend	Cat# 505007	1.0 µg per 10^6 cells in 100 µl volume
Anti-mouse CX3CR1, BV421, clone SA011F11	Biolegend	Cat# 149023	0.03 µg per 10^6 cells in 100 µl volume
Anti-mouse I-A/I-E, PerCP/Cy5.5, clone M5/114.15.2	Biolegend	Cat# 107626	0.06 µg per 10^6 cells in 100 µl volume

Anti-mouse F4/80, FITC, clone BM8	Biolegend	Cat# 123108	0.25 µg per 10^6 cells in 100 µl volume
Anti-mouse CD3, BV605, clone 17A2	Biolegend	Cat# 100237	5 µl per 10^6 cells in 100 µl volume
