# Supplementary material for: Organoids transplantation attenuates intestinal ischemia/reperfusion injury in mice through L-Malic acid-mediated M2 macrophage polarization
Source: Nat Commun. 2023 Oct 25;14:6779. doi: 10.1038/s41467-023-42502-0 (PMC10600233; doi:10.1038/s41467-023-42502-0)
Supplement: Supplementary file 3 — Description of Additional Supplementary Files [file 41467_2023_42502_MOESM3_ESM.pdf]

<u>Description of Additional Supplementary Files</u>

Supplementary Data 1: organoids conditioned medium-positive ion mode

Supplementary Data 2: organoids conditioned medium-negative ion mode

Supplementary Data 3: difference cecal contentsnegative ion mode

Supplementary Data 4: difference cecal contentspositive ion mode
