# Supplementary material for: Organoids transplantation attenuates intestinal ischemia/reperfusion injury in mice through L-Malic acid-mediated M2 macrophage polarization
Source: Nat Commun. 2023 Oct 25;14:6779. doi: 10.1038/s41467-023-42502-0 (PMC10600233; doi:10.1038/s41467-023-42502-0)
Supplement: Supplementary file 8 — Reporting Summary [file 41467_2023_42502_MOESM8_ESM.pdf]

Corresponding author(s): NCOMMS-23-04575B

Last updated by author(s): Sep 28, 2023

Reporting Summary

Nature Portfolio wishes to improve the reproducibility of the work that we publish. This form provides structure for consistency and transparency in reporting. For further information on Nature Portfolio policies, see our Editorial Policies and the Editorial Policy Checklist.

Statistics

For all statistical analyses, confirm that the following items are present in the figure legend, table legend, main text, or Methods section.

n/a	Confirmed	
☐	☒	The exact sample size (*n*) for each experimental group/condition, given as a discrete number and unit of measurement
☐	☒	A statement on whether measurements were taken from distinct samples or whether the same sample was measured repeatedly
☐	☒	The statistical test(s) used AND whether they are one- or two-sided<br>*Only common tests should be described solely by name; describe more complex techniques in the Methods section.*
☒	☐	A description of all covariates tested
☐	☒	A description of any assumptions or corrections, such as tests of normality and adjustment for multiple comparisons
☐	☒	A full description of the statistical parameters including central tendency (e.g. means) or other basic estimates (e.g. regression coefficient) AND variation (e.g. standard deviation) or associated estimates of uncertainty (e.g. confidence intervals)
☐	☒	For null hypothesis testing, the test statistic (e.g. *F*, *t*, *r*) with confidence intervals, effect sizes, degrees of freedom and *P* value noted<br>*Give P values as exact values whenever suitable.*
☒	☐	For Bayesian analysis, information on the choice of priors and Markov chain Monte Carlo settings
☒	☐	For hierarchical and complex designs, identification of the appropriate level for tests and full reporting of outcomes
☐	☒	Estimates of effect sizes (e.g. Cohen's *d*, Pearson's *r*), indicating how they were calculated

Our web collection on statistics for biologists contains articles on many of the points above.

Software and code

Policy information about availability of computer code

Data collection	The data were collected with various instrumentations and equipments such as microscope (Image D2), spectrophotometer (Nano Drop Onec), ABI Q6 Real-Time PCR System (Applied Biosystems), in vivo imaging system (Ami HTX Optical Imaging System), TSQ Quantiva™ (Thermo Fisher Scientific), CBot (HiSeq2500). All other data collection details are discussed in the Methods section.
Data analysis	Graph Pad Prism (version 8.3.0), Image J (version 1.52), ANALYSIS ONLY Aura 4.0.7M, FlowJo (version 10), TraceFinder™ software (version 3.3 SP1), Fast QC (version 0.10.1) .

For manuscripts utilizing custom algorithms or software that are central to the research but not yet described in published literature, software must be made available to editors and reviewers. We strongly encourage code deposition in a community repository (e.g. GitHub). See the Nature Portfolio guidelines for submitting code & software for further information.

Data

Policy information about availability of data

All manuscripts must include a data availability statement. This statement should provide the following information, where applicable:

- Accession codes, unique identifiers, or web links for publicly available datasets
- A description of any restrictions on data availability
- For clinical datasets or third party data, please ensure that the statement adheres to our policy

All data are available in the main text or the supplementary materials. The differentially expressed raw metabolomic data have been provided in Supplementary

Data. The raw RNA–seq data for this study have been deposited in the Sequence Read Archive (SRA) under accession code SRP434172 (https://www.ncbi.nlm.nih.gov/sra/?term=Srp434172). Source data are provided with this paper.

Human research participants

Policy information about studies involving human research participants and Sex and Gender in Research.

Reporting on sex and gender	There was no sex and gender analysis in this study.
Population characteristics	Blood and fecal samples were collected from participants undergoing coronary artery bypass graft or elective cardiac valve replacement surgery.
Recruitment	Patients were consecutively recruited and all participants were aged between 18 and 75 years. Exclusion criteria included those with chronic digestive system diseases, previous gastrointestinal surgery, chronic kidney disease, confirmed or suspected intestinal ischemia/necrosis, and those who had used prebiotics, laxatives or antidiarrheals within one week, or antibiotics within three months before the start of the study.
Ethics oversight	The study was approved by the ethic committee of Nanfang Hospital of Southern Medical University (approval number: NFEC-202009-k2-01)

Note that full information on the approval of the study protocol must also be provided in the manuscript.

Field-specific reporting

Please select the one below that is the best fit for your research. If you are not sure, read the appropriate sections before making your selection.

☒ Life sciences ☐ Behavioural & social sciences ☐ Ecological, evolutionary & environmental sciences

For a reference copy of the document with all sections, see nature.com/documents/nr-reporting-summary-flat.pdf

Life sciences study design

All studies must disclose on these points even when the disclosure is negative.

Sample size	We have adopted at least n=3 biological replicates to calculate the statistical value of each analysis based on the previous publications in the filed (Cell. 2020;180(6):1198-1211.e19.).
Data exclusions	No data were excluded from analysis.
Replication	All experiments were performed in this manuscript at least there times independent biological replicates. All attempts at replication were successful.
Randomization	For in vivo and in vitro studies, samples were randomly assigned to the control group or the experimental group.
Blinding	The investigators were blinded to group allocation during data collection and analysis.

Reporting for specific materials, systems and methods

We require information from authors about some types of materials, experimental systems and methods used in many studies. Here, indicate whether each material, system or method listed is relevant to your study. If you are not sure if a list item applies to your research, read the appropriate section before selecting a response.

Materials & experimental systems

n/a	Involved in the study	
☐	☒	Antibodies
☒	☐	Eukaryotic cell lines
☒	☐	Palaeontology and archaeology
☐	☒	Animals and other organisms
☒	☐	Clinical data
☒	☐	Dual use research of concern

Methods

n/a	Involved in the study	
☒	☐	ChIP-seq
☐	☒	Flow cytometry
☒	☐	MRI-based neuroimaging

Antibodies

Antibodies used	Anti-mouse CD16/32, clone 2.4G2 BD Biosciences Cat# 553141<br>Anti-mouse CD11b, BV510, clone M1/70 Biolegend Cat# 101263<br>Anti-mouse CD11b, PE, clone M1/70 Biolegend Cat# 101208<br>Anti-mouse Ly-6G, PerCP/Cy5.5, clone 1A8 Biolegend Cat# 127615<br>Anti-mouse CD45, APC-Cy7, clone I3/2.3 Thermo Fisher Scientificic Cat# A15395<br>Anti-mouse Ly-6C, PE-Cy7, clone HK1.4 Biolegend Cat# 128017<br>Anti-mouse CD206, APC, clone C068C2 Biolegend Cat# 141707<br>Anti-mouse IL10, PE, clone JES5-16E3 Biolegend Cat# 505007<br>Anti-mouse CX3CR1, BV421, clone SA011F11 Biolegend Cat# 149023<br>Anti-mouse I-A/I-E, PerCP/Cy5.5, clone M5/114.15.2 Biolegend Cat# 107626<br>Anti-mouse F4/80, FITC, clone BM8 Biolegend Cat# 123108<br>Anti-mouse CD3, BV605, clone 17A2 Biolegend Cat# 100237<br>Anti-mouse ZO-1, Abcam Cat# ab216880<br>Anti-mouse occludin, Abcam Cat# ab216327<br>Anti-mouse Ki67, Abcam Cat# ab279653<br>Anti-mouse OLFM4, Cell Signaling Technology Cat# 39141S<br>Anti-mouse Muc2, Proteintech Cat# 27675-1-AP<br>Anti-mouse lysozyme, Dako Cat# A0099<br>Anti-mouse mannose receptor, Abcam Cat# ab64693<br>Chicken anti-GFP antibody Abcam Cat# ab13970<br>FITC-conjugated donkey anti-Chicken antibody Abcam Cat# ab63507
Validation	The antibody used in our experiments is commercially available and has been validated by the manufacturer. All validation statements can be found on the respective antibody website.

Animals and other research organisms

Policy information about studies involving animals; ARRIVE guidelines recommended for reporting animal research, and Sex and Gender in Research

Laboratory animals	All mice included in this study were on a C57BL/6J genetic backgroud. C57BL/6J mice were provided by the Animal Center at the Nanfang Hospital of Southern Medical University (Guangzhou, China). LGR5-EGFP-IRES-CreERT2 (LGR5-GFP) mice were obtained from Jackson Laboratory (Ban Harbor, ME, USA). Breeding pairs of SOCS2 knocked-out mice were kindly provided by Zai-Long Chi (Wenzhou Medical University). SOCS2–/– mice in the C57BL/6 genetic background were generated by using the CRISPR/Cas9-mediated genome engineering technology. The deletion of the exon 3 fragment was carried out using gRNA1 (TTG GCA GTC GTT TTT CTA GT CGG) and gRNA2 (ATT CAG CTA AAA CTA CCT AA GGG) generated by Cyagen Biosciences (Guangzhou, China). All mice included in this study were housed as five mice in a cage under specific pathogen-free (SPF) conditions (12 h light-dark cycle, temperature: 22 ± 1°C and humidity: 55 ± 5%) and had ad libitum access to standard mouse chow (MD17121, MEDICIENCE, Jiangsu, China) and water.
Wild animals	No wild animal has been used in the study.
Reporting on sex	All animals used in this study were male, and no sex-based analysis was performed.
Field-collected samples	The study did not involve samples from the field.
Ethics oversight	Approval of the animal study was obtained from the Nanfang Hospital animal ethics committee (approval number: IACUC-LAC-20220508-001).

Note that full information on the approval of the study protocol must also be provided in the manuscript.

Flow Cytometry

Plots

Confirm that:

☒ The axis labels state the marker and fluorochrome used (e.g. CD4-FITC).

☒ The axis scales are clearly visible. Include numbers along axes only for bottom left plot of group (a 'group' is an analysis of identical markers).

☒ All plots are contour plots with outliers or pseudocolor plots.

☒ A numerical value for number of cells or percentage (with statistics) is provided.

Methodology

Sample preparation	The sample preparation was described in the methods section.

Instrument	All data were obtained with LSRFortessa X-20 Multidimensional HD Flow Cytometer.
Software	The results were analysed using Flow Jo software 10.
Cell population abundance	Minimum of 5000 cells were counted for each analysis.
Gating strategy	Positive populations were determined by the specific antibodies, which were distinct from negative populations.

☐ Tick this box to confirm that a figure exemplifying the gating strategy is provided in the Supplementary Information.
